# Estimating long COVID-19 prevalence across definitions and forms of sample selection

**DOI:** 10.3389/fepid.2025.1597799

**Published:** 2025-05-30

**Authors:** Pietro Giorgio Lovaglio, Fabio Borgonovo, Alessandro Manzo Margiotta, Mohamed Mowafy, Marta Colaneri, Alessandra Bandera, Andrea Gori, Amedeo Ferdinando Capetti

**Affiliations:** ^1^ Department of Statistics and Quantitative Methods, University of Milano Bicocca, Milan, Italy; ^2^ Division of Infectious Diseases, Luigi Sacco Hospital, University of Milan, Milan, Italy; ^3^ Department of Biomedical and Clinical Sciences, University of Milan, Milan, Italy; ^4^ Department of Pathophysiology and Transplantation, University of Milan, Milan, Italy; ^5^ Infectious Diseases Unit, IRCCS Ca' Granda Ospedale Maggiore Policlinico Foundation, Milan, Italy

**Keywords:** long COVID, incidence, risk factors, selection bias, clinical research

## Abstract

**Introduction:**

Long COVID (LC) is a multisystem condition with prolonged symptoms persisting beyond acute SARS-CoV-2 infection. However, prevalence estimates vary widely due to differences in case definitions and sampling methodologies. This study aims to determine the prevalence of LC across different definitions and correct for selection bias using advanced statistical modeling.

**Methods:**

We conducted a retrospective, observational study at Luigi Sacco Hospital (Milan, Italy), analyzing 3,344 COVID-19 patients from two pandemic waves (2020–2021). Participants included 1,537 outpatients from the ARCOVID clinic and 1,807 hospitalized patients. LC was defined based on WHO and NICE criteria, as well as two alternative definitions: symptoms persisting at 3 and 6 months post-infection. We used a bivariate censored Probit model to account for selection bias and estimate adjusted LC prevalence.

**Results:**

LC prevalence varied across definitions: 67.4% (WHO), 76.3% (NICE), 80.2% (3 months), and 79.6% (6 months). Adjusted prevalence estimates remained consistent across definitions. The most common symptoms were fatigue (58.6%), dyspnea (41.1%), and joint/muscle pain (39.2%). Risk factors included female sex (OR 2.165–2.379), metabolic disease (OR 1.587–1.629), and older age (40–50 years, OR 1.847). Protective factors included antiplatelets (OR 0.640–0.689), statins (OR 0.616), and hypoglycemics (OR 0.593–0.706). Vaccination, hydroxychloroquine, and antibiotics were associated with an increased risk of LC. Selection bias significantly influenced prevalence estimates, underscoring the need for robust statistical adjustments.

**Discussion:**

Our findings highlight the high prevalence of LC, particularly among specific subgroups, with strong selection effects influencing outpatient participation. Differences in prevalence estimates emphasize the impact of case definitions and study designs on LC research. The identification of risk and protective factors supports targeted interventions and patient management strategies.

**Conclusion:**

This study provides one of the most comprehensive analyses of LC prevalence while accounting for selection bias. Our findings call for standardized LC definitions, improved epidemiological methodologies, and targeted prevention strategies. Future research should explore prospective cohorts to refine LC prevalence estimates and investigate long-term health outcomes.

## 1 Introduction

Long COVID (LC) is a multisystem disease that has a devastating effect on almost every organ system (1), with potentially lifelong consequences. The World Health Organization (WHO) has defined the condition as the continuation or development of new symptoms 3 months after from the initial SARS-CoV-2 infection (2). Symptoms must last at least 2 months and lack alternative explanation.

The UK Office for National Statistics estimated that, in 2022, self-reported LC at a population level was 2.7% (3). In the same year, 6.9% of U.S. adults reported ever experiencing LC, and the prevalence ranged from 1.9% to 10.6% (4). Global estimates suggest 65 million people now suffer from LC (5). According to the WHO definition, more than 17 million people across the WHO European Region may have experienced it during the first two years of the pandemic (2020/21) and the percentage of people with LC should range from 10 to 20% (6).

Rates of LC among people who have contracted SARS-CoV-2 vary controversially between studies and regions, from about 10 to 50% (7). A systematic review examining the frequency and variety of persistent symptoms within 60 days of COVID-19 onset reported that the median proportion of patients with at least one persistent symptom was 72.5% (8).

Two important studies estimated the prevalence of LC using a control group to compare the presence and severity of symptoms before and after COVID-19 and assessed the LC status based on the onset of symptoms, 6 months from the acute phase (9) vs. 3–5 months (10).

In a nationwide population cohort study of 198,096 Scottish adults (9), the crude prevalence of one or more symptoms attributable to SARS-CoV-2 infection ranged from 13.2 to 14.3% at 6 months, reduced to 6.3%–6.9% following adjustment for potential confounders. A similar study designed in the Netherlands (10) suggested a 12.7% higher prevalence of symptoms at 3–5 months after COVID-19 infection (21.4%) vs. COVID-19-negative controls (8.7%).

In this end rarely studies of LC among infected patients include an uninfected control group or take into account selection biases studying a wider population that -for different unknown reasons- did not take part in the study (LC follow-up).

In the present paper, we aim to analyse the prevalence of LC, after SARS-CoV-2 infection, and the severity of long-term symptoms related to COVID-19.

## 2 Methods

### 2.1 Study design and clinical setting

This retrospective, observational, single centre study was conducted at Luigi Sacco Hospital (Milan, Italy) between May 2020 and July 2021. The hospital, a designated COVID-19 referral centre in northern Italy, established the ARCOVID outpatient clinic (“Ambulatorio Rivalutazione COVID-19”) to monitor patients recovering from SARS-CoV-2 infection.

### 2.2 Study population

The medical records of non-hospitalized patients were prospectively collected. Inclusion criteria included patients aged over 18 years with a confirmed diagnosis of COVID-19, established through PCR testing or detection of anti-N antibodies. Following the provision of written informed consent, eligible patients were enrolled in a longitudinal clinical study.

Participants were referred to the ARCOVID outpatient clinic either by specialists, general practitioners, or through self-referral.

### 2.3 Participants group

The study included patients who experienced COVID-19 during Italy's first two pandemic waves: Wave 1 (February 21–May 31, 2020) and Wave 2 (October 1, 2020–July 31, 2021), over a follow-up period of approximately 30 months (April 29, 2020, to October 4, 2022). Clinical history was reviewed during the initial visit and classified using the WHO COVID-19 severity score (mild, moderate, severe, critical) (11). A standardized clinical evaluation was conducted, including a 6-minute walk test and thoracic ultrasound in cases of dyspnea. Persistent symptoms were assessed using standardized questions on 11 symptoms: palpitations, memory impairment, headache, anxiety/panic, insomnia, loss of smell, loss of taste, dyspnea, fatigue, muscle pain, and telogen effluvium. This symptom set was derived from an internal, patient-driven survey performed in our post-COVID clinic before the publication of any formal LC definitions; these 11 manifestations emerged as the most frequently reported and were therefore targeted for systematic evaluation. Patients were referred to specialists as needed based on their clinical presentation.

### 2.4 Control group

The control group consisted of all patients hospitalized during the first two waves of COVID-19 at Luigi Sacco Hospital. Data for this group were obtained from the hospital discharge database and clinical records.

### 2.5 Follow up

The follow-up of the participant group was conducted through email questionnaires sent every three months to monitor symptoms and overall health status. Patients without email access were contacted by phone and invited to provide their responses on paper.

### 2.6 Case definitions

LC was defined according to WHO and National Institute for Health and Care Excellence (NICE) definition (2, 12). Unlikely the WHO definition, the NICE definition of LC includes both ongoing symptomatic COVID-19 (5–12 weeks after onset) and post-COVID-19 Syndrome (12 weeks or more).

Prevalence of LC were estimated, apart the Who definition (LC_WHO) and NICE definition (LC_NICE), as well as, for two alternative definitions that were utilized in the medical literature, such as the appearance of at least one symptom 3 (LC_3 m) and 6 (LC_6 m) months after SARS-CoV-2 infection for at least one symptom, ignoring their duration.

### 2.7 Outcomes

The primary objective of this study was to analyse the prevalence of LC following SARS-CoV-2 infection and to assess the severity of long-term symptoms associated with COVID-19.

### 2.8 Ethics

All participants gave their written informed consent for inclusion in the study, which was conducted in accordance with the Declaration of Helsinki. The study protocol received approval from the Institutional Review Board of Luigi Sacco Hospital (“Comitato etico aziendale”).

### 2.9 Available Data

For all included patients, demographic information such as age and gender were collected. Clinical data encompassed details about comorbidities, including obesity, respiratory diseases, cardiovascular diseases, metabolic disorders, onco-hematologic conditions, immune system disorders, hepatic diseases, renal diseases and diabetes. Additionally, the history of previous COVID-19 infection was recorded and classified based on the WHO COVID-19 severity scale. The vaccination status of each patient was also documented to provide insight into their immunization history against SARS-CoV-2.

History of previous COVID19 was also recorded and classified through COVID-19 Severity (WHO scale) (11).

The need for oxygen therapy during the acute phase of COVID-19 was recorded and categorized into six specific levels: no oxygen therapy; low-flow oxygen via nasal cannula; moderate oxygen support via a Venturi mask; high-flow oxygen via a reservoir mask; advanced respiratory support with non-invasive ventilation (e.g., CPAP or BiPAP); and tracheal intubation with invasive mechanical ventilation.

Medications administered during the acute phase were also documented and included antiplatelet agents, statins, hypoglycemic drugs, antiretrovirals, hydroxychloroquine, and antibiotics.

Post-acute symptoms were systematically assessed, focusing on a range of issues such as palpitations, memory impairment, headache, anxiety or panic, insomnia, loss of smell, loss of taste, dyspnea, fatigue, muscle pain, and telogen effluvium.

### 2.10 Exclusion restrictions

Exclusion restrictions (ER) are variables that influence study participation but do not directly affect the development of LC, making their selection a critical methodological consideration. Fortunately, the extensive research on LC provides valuable guidance. A recent systematic review and meta-analysis of 41 studies identified key clinical and non-clinical risk factors associated with post-COVID-19 syndrome (13). The findings indicate that female sex, older age, higher body mass index, smoking, prior hospitalization or ICU admission, and preexisting conditions, particularly cardiovascular disease, metabolic disorders, and diabetes are significantly associated with an increased risk of LC.

Conversely, immune system disorders, pulmonary diseases, renal disease, and cancer were either not consistently identified as LC risk factors or lacked sufficient evidence due to study variability. While these conditions may influence healthcare access and study participation, current research does not support a direct causal link to LC. Therefore, they will be used as exclusion restrictions (ER) solely in the selection equation to improve model validity and minimize confounding.

### 2.11 Statistical analysis

To deal with sample selection bias we use a bivariate binary selection model. Specifically, we adopted a bivariate censored Probit (14, 15), that jointly models two binary variables, one for the selection equation (y1) or ARCOVID participation and one for the binary outcome equation (y2).

More specifically, the bivariate censored Probit suitably models the bivariate observation mechanism in our application: we observe yi2 (=1 if subject presents LC) if and only if yi1 = 1 (=1 if subject i is ARCOVID participant), whereas if yi1 = 0, we lack information about yi2 (also known as, non-ignorable missing responses). Thus, the first Probit equation is completely observed (selection equation), but we have only a selected (censored) sample for the second equation (outcome equation).

The bivariate Probit model, where equations' errors follow a jointly Normal bivariate distribution with an association parameter r, was specified using k and p observed covariates Z1 and Z2 for each equation, respectively, also specifying covariates appearing in the selection equation, but not the outcome equation (exclusion restrictions, ERs), for correct identification of the bivariate sample selection model.

The significance of r indicates the amount of selection biases in the selected sample (or the biases remaining after controlling for observed covariates Z1 and Z2, thus the bias due to unobserved confounders) and determines whether the two equations should be estimated jointly (when the r estimate is significant) or, in the opposite case, separately.

More specifically, our primary outcome was LC prevalence, P (yi2 = 1).

Prevalence of LC were estimated for LC_WHO, LC_3 m and LC_6 m (LC_NICE was not modelled, but we report only the raw estimate).

For each LC definition, we estimate for ARCOVID participants, the crude attributable prevalence (naïve prevalence) and the corrected version by the bivariate Probit model that adjust for observed and unobserved confounders in the outcome equation and participation equation (adjusted prevalence).

Estimates of naïve and adjusted prevalence were calculated for the whole LC condition (at least one symptom), and then by each symptom (LC condition of each symptom). Other details can be found in the Statistical Appendix.

## 3 Results

The study analysed a total of 3,344 patients, of whom 1,537 (46%) attended the ARCOVID outpatient clinic and 1,807 (54%) were hospitalized during the first two waves of COVID-19 at our hospital (Table 1). Participants were generally younger than controls, with 65.4% aged > 50 compared to 78.3% in the control group. Participation in the ARCOVID outpatient program decreased with age: individuals aged 41–50 and 51–102 were 41% and 64% less likely to participate, respectively, than those aged 18–30. Females were more likely to participate (OR 1.706). Pre-existing health conditions significantly affected participation. Patients with oncological diseases had over an 80% reduction in participation odds. Each additional comorbidity decreased participation likelihood by nearly 25%. The control group had a higher average number of comorbidities (mean 1.66, SD 1.40) compared to the participant group (mean 1.17, SD 1.25).

**Table 1:** Counts, percentages and odds ratio (OR, treated vs. control) and OR significance (P).

Covariates	Levels	Controls	Participants	Overall	OR	*P*
		(*N* = 1,807)	(*N* = 1,537)	(*N* = 3,344)		
Age Group	[18, 30)	64 (3.5%)	125 (8.1%)	189 (5.7%)		
	[30, 40]	104 (5.8%)	147 (9.6%)	251 (7.5%)	0.724	
	(40, 50]	225 (12.5%)	260 (16.9%)	485 (14.5%)	0.592	***
	(50, 102]	1,414 (78.3%)	1,005 (65.4%)	2,419 (72.3%)	0.364	***
Sex	Male	1,110 (61.4%)	742 (48.3%)	1,852 (55.4%)		
	Female	697 (38.6%)	795 (51.7%)	1,492 (44.6%)	1.706	***
Obesity	0	1,381 (76.4%)	1,283 (83.5%)	2,664 (79.7%)		
	1	426 (23.6%)	254 (16.5%)	680 (20.3%)	0.642	***
Lung	0	1,544 (85.4%)	1,364 (88.7%)	2,908 (87.0%)		
	1	263 (14.6%)	173 (11.3%)	436 (13.0%)	0.745	***
Heart	0	889 (49.2%)	989 (64.3%)	1,878 (56.2%)		
	1	918 (50.8%)	548 (35.7%)	1,466 (43.8%)	0.537	***
Metabolic	0	1,233 (68.2%)	1,150 (74.8%)	2,383 (71.3%)		
	1	574 (31.8%)	387 (25.2%)	961 (28.7%)	0.723	***
Renal	0	1,657 (91.7%)	1,477 (96.1%)	3,134 (93.7%)		
	1	150 (8.3%)	60 (3.9%)	210 (6.3%)	0.449	***
Oncological	0	1,590 (88.0%)	1,493 (97.1%)	3,083 (92.2%)		
	1	217 (12.0%)	44 (2.9%)	261 (7.8%)	0.216	***
Immune System	0	1,675 (92.7%)	1,448 (94.2%)	3,123 (93.4%)		
	1	132 (7.3%)	89 (5.8%)	221 (6.6%)	0.780	*
Hepatopathy	0	1,738 (96.2%)	1,510 (98.2%)	3,248 (97.1%)		
	1	69 (3.8%)	27 (1.8%)	96 (2.9%)	0.450	***
Diabetes	0	1,559 (86.3%)	1,397 (90.9%)	2,956 (88.4%)		
	1	248 (13.7%)	140 (9.1%)	388 (11.6%)	0.630	***
#Comorbidities	Mean (Std. Dev.)	1.66 (1.40)	1.17 (1.25)	1.43 (1.36)	0.755	***
	Median [Min, Max]	1.00 [0, 7.00]	1.00 [0, 7.00]	1.00 [0, 7.00]		

Sign levels: *p < 0.1; **p < 0.05; ***p < 0.01.

Table 2 shows that in the acute phase of COVID-19, 42.3% of participants were asymptomatic, compared to 14.2% of the control group. The second largest participant group required oxygen therapy for lung insufficiency (23.3%), followed by those with extrapulmonary involvement (22.1%) and lung insufficiency without oxygen therapy (12.4%). Over half of the participants did not require oxygen therapy, compared to only 19.6% of the control group.

**Table 2:** Counts, percentages and odds ratio (OR, treated vs. control) and OR significance (P).

Covariates	Controls	Participants	Overall	OR	*P*
	(*N* = 1,807)	(*N* = 1,537)	(*N* = 3,344)		
COVID-19 Severity (WHO scale)					
1- Asymptomatic COVID-19	256 (14.2%)	650 (42.3%)	906 (27.1%)	–	
2- Extrapulmonary COVID-19	811 (44.9%)	339 (22.1%)	1,150 (34.4%)	0.165	***
3- COVID-19 + lung insufficiency	490 (27.1%)	190 (12.4%)	680 (20.3%)	0.153	***
4- COVID-19 + lung insufficiency + O2 support	250 (13.8%)	358 (23.3%)	608 (18.2%)	0.564	***
O2 Therapy during COVID-19					
1- NO O2 therapy	355 (19.6%)	818 (53.2%)	1,173 (35.1%)	–	
2- Nasal cannula	445 (24.6%)	193 (12.6%)	638 (19.1%)	0.188	***
3- Venturi mask	410 (22.7%)	196 (12.8%)	606 (18.1%)	0.207	***
4- Reservoir mask	75 (4.2%)	29 (1.9%)	104 (3.1%)	0.168	***
5- Non-invasive ventilation	357 (19.8%)	240 (15.6%)	597 (17.9%)	0.292	***
6- IOT (Tracheal intubation)	165 (9.1%)	61 (4.0%)	226 (6.8%)	0.160	***

O2 Therapy: Nasal cannula: low O2 flow; Venturi mask: 28–60% O2 flow; Reservoir mask: 100% O2 flow; Non-invasive ventilation: 100% O2 flow.

Sign levels: *p < 0.1; **p < 0.05; ***p < 0.01.

Table 3 presents the crude prevalence of experiencing at least one LC symptom within 3 and 6 months after the onset of acute COVID-19. It also reports the crude prevalence of LC as defined by the WHO and the NICE. The participation rate was 53% during the first wave and 43% during the second wave of the COVID-19 pandemic. Overall, participants from the first and second waves accounted for 31% and 69% of the total study population, respectively.

**Table 3:** Upper: ARCOVID participants (N) and prevalence of long COVID-19 by definition.

Covariates	Overall period	Wave1	Wave2	*P* **^^^**
	*N* = 1,537	*N* = 476	*N* = 1,061	
Long COVID-19	N (Prev.)			
LC_WHO	1,036 (67.4%)	342 (72.0%)	694 (65.3%)	<0.001
LC_Nice	1,173 (76.3%)	371 (78.1%)	802 (75.5%)	<0.001
LC_3 m	1,232 (80.2%)	383 (80.6%)	849 (79.9%)	<0.001
LC_6 m	1,223 (79.6%)	379 (79.8%)	844 (79.5%)	<0.001
Single symptom	N (Prev.)			
Dyspnea	631 (41.1%)	185 (38.9%)	446 (42.0%)	<0.001
Hair loss	405 (26.3%)	140 (29.5%)	265 (24.9%)	<0.001
Fatigue	900 (58.6%)	286 (60.2%)	614 (57.8%)	<0.001
Join Muscle Pain	602 (39.2%)	202 (42.5%)	400 (37.7%)	<0.001
Palpitations	383 (24.9%)	130 (27.4%)	253 (23.8%)	<0.001
Smell Loss	334 (21.7%)	140 (29.5%)	194 (18.3%)	<0.001
Taste Loss	322 (20.9%)	133 (27.9%)	189 (17.8%)	0.001
Headaches	299 (19.5%)	104 (21.9%)	195 (18.4%)	<0.001
Anxiety	352 (22.9%)	116 (24.4%)	236 (22.2%)	<0.001
Insomnia	396 (25.8%)	96 (20.2%)	300 (28.2%)	<0.001

Lower: ARCOVID Participants (N) and Prevalence (Prev.) of single symptoms, overall period by wave.

LC, long Covid.

^^^ Significance of differences of prevalence across waves.

The majority of patients experienced LC, with prevalence estimates ranging from 67.4%, according to WHO definition, to 80.2% within 3 months of initial infection. Significant difference in LC prevalence between the two waves were found, with a generally lower prevalence observed in the second wave. The most prevalent symptoms were fatigue (58.6%), dyspnea (41.1%), and joint and muscle pain (39.2%).

Table 4 presents the naïve and adjusted prevalence of LC alongside the results of the selection equation from the bivariate probit model, which jointly estimates the likelihood of ARCOVID participation and the probability of experiencing LC under various definitions.

**Table 4:** Upper: naïve and adjusted prevalence by LC definition and significance of the sample selection (Rho) of the bivariate probit model. Lower: covariates and significance (P) of the selection equation.

Outcome	LC_WHO			LC_3 m			LC_6 m		
Naïve Prev. (95% CI)	67.4% (65.1%–69.7%)			80.2% (78.2%–82.1%)			79.6% (77.6%–81.6%)		
Adjusted Prev. (95% CI)	66.1% (50.1%–75.6%)			81.1% (71.3%–86.7%)			79.7% (71.2%–85.3%)		
Rho^T^	0.19 (−0.03; 0.37)			**0.37** (0.11; 0.57)			**0.31** (0.06; 0.49)		
Selection equation									
	Coeff.	OR^⊥^	*P*	Coeff.	OR^⊥^	P	Coeff.	*OR^⊥^*	*P*
Constant	0.603	2.671	***	0.796	3.696	***	0.797	3.704	***
Wave (2 vs. 1)				−0.119	0.821	**	−0.119	0.820	**
Oncological	−0.825	0.243	***	−0.824	0.243	***	−0.826	0.243	***
Renal	−0.322	0.579	***	−0.322	0.578	***	−0.323	0.577	***
Lung	−0.105	0.833	.	−0.118	0.816	*	−0.117	0.817	*
Immune system	−0.153	0.763	.	−0.141	0.781	.	−0.146	0.775	
O2_therapy—Nasal cannula	−1.010	0.196	***	−0.991	0.201	***	−0.991	0.201	***
O2_therapy—Venturi mask	−0.932	0.219	***	−0.908	0.229	***	−0.91	0.228	***
O2_therapy—Reservoir mask	−1.083	0.176	***	−1.089	0.174	***	−1.076	0.177	***
O2_therapy—Non invasive ventilation	−0.758	0.292	***	−0.739	0.301	***	−0.736	0.302	***
O2_therapy—IOT (Tracheal intubation)	−1.153	0.152	***	−1.137	0.156	***	−1.138	0.156	***

O2 Therapy: Nasal cannula: low O2 flow; Venturi mask: 28–60% O2 flow; Reservoir mask: 100%O2 flow; Non-invasive ventilation: 100%O2 flow.

^T^ Bold indicates significant sample selection effect (significant Rho estimate).

^⊥^OR estimated using a bivariate Logit model.

Sign levels: “.” *p* < 0.1; “*”*p* < 0.05; “**”*p* < 0.01; “***”*p* < 0.001.

Naïve and adjusted prevalence rates for LC were similar across definitions: 80.2% vs. 81.1% at 3 months, 79.6% vs. 79.7% at 6 months, and 67.4% vs. 66.1% under the WHO definition. The correlation coefficient (Rho) between the error terms of the selection and outcome equations was positive and significant for LC at 3 months [0.37, CI (0.11, 0.57)] and 6 months [0.31, CI (0.06, 0.49)], supporting the use of joint modelling. However, under the WHO definition, the correlation was insignificant [0.19, CI (−0.03, 0.37)], suggesting an absence of selection bias for participants in this context.

The marginal effects from the selection equation of the bivariate probit model highlight the influence of key covariates on the likelihood of ARCOVID outpatient participation. The second wave of COVID-19 was associated with an 18% reduction in participation odds compared to the first wave when modelling LC at 3 and 6 months, but this variable was not significant when modelling LC under the WHO definition.

Model estimates confirm that patients with pre-existing health conditions were significantly less likely to participate in the ARCOVID outpatients ‘clinic. Specifically, those with oncological diseases had 76% lower odds, and those with renal diseases had 42% lower odds of participation across all LC definitions. These effects are strongly significant. In contrast, lung diseases and immune system disorders had less pronounced effects. Lung diseases reduced participation odds by 18% but only in models for LC at 3 and 6 months, while immune system disorders were insignificant in all models.

The severity of the acute COVID-19 phase strongly influenced participation. While higher oxygen therapy intensity did not consistently reduce participation odds, patients requiring any oxygen therapy had 70% to 85% lower odds of participation compared to those who did not, across all LC definitions modelled in the outcome equation.

Table 5 summarizes the coefficients and odds ratios from the outcome equation, estimating the probability of experiencing LC across three definitions.

**Table 5:** Outcome equation for event “long COVID-19” by LC definition: coefficient and significance (P) of the bivariate probit model and odds ratio (OR) of the bivariate logit model.

Covariates	LC_WHO			LC_3 m			LC_6 m		
	Coeff.	*P*	OR^^^	Coeff.	*P*	OR^^^	Coeff.	*P*	OR^^^
Constant	0.048	0.874	1.074	0.432	.	1.942	0.300	.	1.541
Sex Female	0.474	***	2.165	0.510	***	2.351	0.515	***	2.379
Heart disease	0.196	**	1.371						
Metabolic disease				0.310	***	1.629	0.288	***	1.587
Age group [30–40]				0.280	*	1.580	0.237	.	1.482
Age group (40–50]				0.370	**	1.847	0.334	**	1.748
Age group (50–102]				0.263	*	1.520	0.256	*	1.510
Age group [18–30)	Reference			-			-		
Therapies during COVID-19									
Antiaggregant	−0.273	**	0.640	−0.231	*	0.689			
Statin				−0.215	.	0.716	−0.299	**	0.616
Hypoglycaemics	−0.210	.	0.706	−0.310	**	0.602	−0.323	**	0.593
Antiretroviral	−0.541	***	0.410	−0.616	***	0.364	−0.599	***	0.373
HCQ	0.340	***	1.752	0.236	*	1.491	0.251	**	1.540
Antibiotic	0.235	***	1.474	0.175	**	1.336	0.178	**	1.356
Vaccine	0.094	.	1.167	0.137	**	1.254	0.143	**	1.270

^^^ Estimated using a bivariate Logit model. Sign levels: “.” *p* < 0.1; “*”*p* < 0.05; “**”*p* < 0.01; “***”*p* < 0.001.

Female gender significantly increased the odds of LC, more than doubling the risk across all definitions. While age was not a predictor under the WHO definition, advancing age increased LC risk at 3- and 6-months post-infection. Individuals aged 40–50 had 85% and 75% greater odds of LC at 3 and 6 months, respectively, compared to those aged 18–30.

Among pre-existing conditions, cardiovascular disease was a significant risk factor under the WHO definition, increasing LC odds by 37%. However, this association was absent for LC at 3 and 6 months, where metabolic disease emerged as a significant predictor, raising LC odds by 63% and 59%, respectively.

Regarding medications, pre-COVID use of antiaggregant significantly reduced LC odds, by 73% under the WHO definition and by 77% at 3 months. Statins were protective at 6 months, lowering LC odds by nearly 40%. Hypoglycaemic agents consistently reduced LC risk across all definitions, decreasing odds by 30% under the WHO definition and by 40% at 3 and 6 months.

Use of antiretroviral therapy during acute COVID-19 was protective, reducing LC odds by 59%–63% across all definitions. Conversely, hydroxychloroquine (HCQ), antibiotics, and vaccination were identified as risk factors, showing similar effects across all LC definitions.

Tables 6–8 details the prevalence of symptoms under the WHO definition and associated with LC at 3 and 6 months. It includes both observed (naïve) and adjusted prevalence derived from the bivariate probit model, along with the residual sample selection effect (Rho) after accounting for covariates and risk factors. Symptom-specific prevalence was generally lower than overall LC prevalence. Fatigue was the most prevalent symptom across all definitions, while headache-related LC had the lowest prevalence under the WHO definition and at 3 months. Hair loss had the lowest prevalence at 6 months, indicating that headaches may emerge later as an LC symptom, whereas hair loss diminishes by 6 months.

**Table 6:** Naïve and adjusted symptoms’ LC prevalence (and 95% confidence interval) and estimated sample selection effect (Rho) by long COVID-19 (LC) definition.

LC_WHO	Prev. naïve (95% CI)	Rho^^^ (95% CI)	Prev. adjusted^^^^ (95% CI)
LC_Dyspnea	31.1 (28.8–33.4)	**0.291** (0.084, 0.533)	24.2 (20.3–28.7)
LC_Hair_loss	21.9 (19.9–24.0)	0.188 (−0.031, 0.386)	18.2 (14.9–22.4)
LC_Fatigue	47.0 (44.5–49.5)	0.183 (−0.007, 0.341)	41.6 (37.0–47.4)
LC_Joint Muscle Pain	31.8 (29.4–34.1)	0.093 (−0.097, 0.306)	29.3 (23.8–34.2)
LC_Palpitations	20.2 (18.2–22.2)	−0.376 (−0.557, −0.216)	30.1 (23.1–35.9)
LC_Smell Loss	16.1 (14.2–17.9)	−0.481 (−0.601, −0.312)	28.4 (22.8–34.2)
LC_Taste Loss	14.7 (12.9–16.5)	−0.450 (−0.600, −0.308)	25.6 (20.2–32.9)
LC_Amnesia	28.6 (26.4–30.9)	−0.174 (−0.345, 0.013)	33.4 (26.6–40.7)
LC_Headaches	14.6 (12.8–16.3)	−0.409 (−0.586, −0.190)	24.2 (18.1–30.8)
LC_Anxiety	19.5 (17.5–21.4)	−0.029 (−0.242, 0.214)	20.1 (15.9–26.4)
LC_Insomnia	25.6 (23.4,27.8)	−0.110 (−0.332, 0.075)	28.4 (21.2,33.7)

LC, long Covid.

^^^ Bold indicates significant (residual) sample selection effect (significant Rho estimate).

^^^^ Estimated in a bivariate Probit model with only significant (symptom-specific) covariates.

**Table 7:** Naïve and adjusted symptoms’ LC prevalence (and 95% confidence interval) and estimated sample selection effect (Rho) by long COVID-19 (LC) definition.

LC_3 m	Prev. naïve (95% CI)	Rho**^^^** (95% CI)	Prev. adjusted**^^^^** (95% CI)
LC_Dyspnea	42.5 (39.7–45.2)	**0.412** (0.205, 0.568)	31.8 (27.2–36.0)
LC_Hair_loss	23.8 (21.2–26.1)	**0.254** (0.032, 0.482)	19.5 (16.2–24.0)
LC_Fatigue	55.9 (52.8–59.0)	**0.195** (0.025, 0.398)	55.2 (49.3–61.6)
LC_Joint Muscle Pain	36.4 (33.8–38.9)	0.082 (−0.105, 0.271)	39.5 (33.0–45.2)
LC_Palpitations	26.2 (23.9–28.4)	−0.379 (−0.531, −0.199)	33.4 (28.2–39.5)
LC_Smell Loss	19.9 (17.7–22.1)	−0.510 (−0.645, −0.368)	39.1 (33.0–45.2)
LC_Taste Loss	18.7 (16.6–20.9)	−0.489 (−0.624, −0.253)	37.0 (32.1–43.0)
LC_Amnesia	34.5 (31.2–37.8)	−0.126 (−0.289, 0.190)	33.6 (28.7–39.5)
LC_Headaches	17.8 (15.4–20.1)	−0.386 (−0.548, −0.232)	32.4 (26.8–38.7)
LC_Anxiety	21.4 (19.1–23.6)	0.058 (−0.196, 0.316)	23.7 (19.1–29.5)
LC_Insomnia	28.1 (25.3–30.4)	−0.086 (−0.267, 0.142)	26.9 (21.5–33.2)

LC, long Covid.

^^^ Bold indicates significant (residual) sample selection effect (significant Rho estimate).

^^^^ Estimated in a bivariate Probit model with only significant (symptom-specific) covariates.

**Table 8:** Naïve and adjusted symptoms’ LC prevalence (and 95% confidence interval) and estimated sample selection effect (Rho) by long COVID-19 (LC) definition.

LC_6 m	Prev. naïve (95% CI)	Rho^^^ (95% CI)	Prev. adjusted^^^^ (95% CI)
LC_Dyspnea	40.6 (38.1–43.1)	**0.390** (0.197, 0.587)	30.1 (25.7–34.6)
LC_Hair_loss	10.5 (9.0–12.1)	−0.099 (−0.379, 0.126)	12.0 (8.2–16.3)
LC_Fatigue	58.1 (55.6–60.6)	0.173 (−0.014, 0.341)	52.9 (45.3–60.6)
LC_Joint Muscle Pain	38.9 (36.5–41.3)	0.057 (−0.144, 0.250)	37.3 (30.5–42.5)
LC_Palpitations	24.5 (22.3–26.6)	−0.400 (−0.536, −0.198)	35.8 (29.5–42.0)
LC_Smell Loss	21.3 (19.3–23.4)	−0.538 (−0.659, −0.370)	37.0 (30.7–42.3)
LC_Taste Loss	20.6 (18.6–22.6)	−0.514 (−0.655, −0.338)	35.3 (30.2–42.0)
LC_Amnesia	32.8 (30.4–35.1)	−0.102 (−0.283, 0.110)	35.7 (29.2–42.3)
LC_Headaches	19.3 (17.3–21.3)	−0.410 (−0.582, −0.265)	30.1 (23.8–35.8)
LC_Anxiety	22.8 (20.7–24.9)	0.034 (−0.182, 0.231)	22.0 (17.1–27.3)
LC_Insomnia	25.6 (23.4–27.8)	−0.110 (−0.269, 0.069)	28.4 (22.8–34.7)

LC, long Covid.

^^^ Bold indicates significant (residual) sample selection effect (significant Rho estimate).

^^^^ Estimated in a bivariate Probit model with only significant (symptom-specific) covariates.

The residual sample selection effect (Rho) was significant for dyspnea, palpitations, smell loss, taste loss, and headaches under the WHO definition and at 6 months. Hair loss and fatigue also showed significant Rho at 3 months. For these symptoms, adjusted prevalence provided key insights: dyspnea-related LC was less common after adjustment, while palpitations, smell loss, taste loss, and headache were more prevalent under the WHO definition and at 6 months. Hair loss and fatigue exhibited higher adjusted prevalence at 3 months.

## 4 Discussion

In our study population of 1,537 participants, the prevalence of LC ranged from 66.1%, based on the WHO definition, to 81.1% when defined as the presence of at least one LC symptom within three months of acute COVID-19. This higher prevalence can be attributed to the unique study setting, which includes both hospitalized patients and individuals seeking care at a clinic for persistent symptoms. Despite variations in LC prevalence depending on the definition used, our findings are consistent with those reported in studies from COVID-19 referral hospitals in the USA and Ireland (16, 17). Population-based studies estimate the prevalence of LC to range between 10% and 20%, as defined by the 2022 WHO criteria (6), supported by several population-based studies (9) and a US-based meta-analysis (2). These proportions shift in meta-analyses that include heterogeneous studies of hospitalized, non-hospitalized, and mixed populations, adopting a more clinical approach with a higher risk of focusing on specific subgroups rather than on the general population. For example, O'Mahoney et al. (18) reported a prevalence of 45% after an average follow-up of four months post-acute COVID-19.

Notably, our results reveal strong selection effects, indicating that patients with pre-existing oncological or renal diseases, as well as those with more severe cases of COVID-19, were less likely to attend the ARCOVID outpatient clinic. This is likely because these patients often returned to their respective referral clinics (e.g., oncological or nephrological) after the acute phase of COVID-19, while those with more severe illness were more frequently referred to pneumological outpatient clinics. Additionally, our estimates do not show a significant wave effect on the likelihood of developing LC between the first two waves, consistent with the findings of Aloe et al. (19).

The study identified several significant risk and protective factors associated with LC. Female gender was a prominent risk factor as already known in literature (20). While age was not a significant predictor under the WHO definition, it was linked to increased odds of LC at 3- and 6-months post-infection, particularly among individuals aged 40–50, who demonstrated significantly higher odds compared to those aged 18–30. This finding contrasts with current knowledge but may be attributed to the underrepresentation of younger patients in our cohort (21).

Among pre-existing health conditions, cardiovascular disease was identified as a significant risk factor for LC under the WHO definition, increasing the odds of its occurrence. However, this association was not observed for LC at 3- and 6-months post-infection, where metabolic disease emerged as a significant risk factor.

This interpretation aligns with current literature and, although cardiovascular disease (CVD) is a well-established risk factor for severe acute COVID-19, its role in LC remains less clear. Several cohort studies have reported links between pre-existing CVD and persistent LC symptoms, but these associations are inconsistent and generally weaker than those observed for metabolic disorders (22, 23).

Loosen et al. highlighted lipid metabolism disorders and obesity as strong, independent risk factors for LC, while the evidence regarding cardiovascular disease remains less robust (24).

Proposed mechanisms for the stronger metabolic signal include chronic, low-grade inflammation, immune dysregulation, and endothelial dysfunction driven by insulin resistance and dyslipidemia, pathways that may more directly perpetuate symptom persistence than those primarily affecting cardiac function (25). By contrast, patients with CVD often receive guideline-based cardioprotective therapies and closer clinical monitoring, which may mitigate long-term sequelae and contribute to the weaker, more variable associations seen in LC cohorts.

The study also underscored the impact of medications on LC outcomes. Antiplatelet medications demonstrated significant protective effect, markedly reducing the odds of LC under the WHO definition and at 3 months post-infection, although evidence supporting their protective role remains limited (26). Similarly, while statins showed protective effects at 6 months, the evidence for their role in LC prevention is also scarce (27). In contrast, the protective role of hypoglycaemic agents, particularly metformin, and antiretroviral medications during the acute phase is already well-recognized, both being consistently associated with a reduced likelihood of experiencing LC (28, 29).

Conversely, the use of hydroxychloroquine and antibiotics were identified as risk factors, exhibiting comparable magnitudes across all definitions of LC. These findings contrast with recent evidence by Pasculli et al. (30), who reported that hydroxychloroquine treatment was associated with a higher risk of chest CT residual lesions in hospitalized patients but did not identify it as a risk factor for developing LC syndrome, and with Brogna et al. (31), who found that early antibiotic therapy significantly shortened recovery time in COVID-19 patients without contributing to the development of LC.

Although, in our study, HCQ and azithromycin have been linked to changes in LC incidence, this association may actually reflect their tendency to worsen underlying cardiometabolic comorbidities, especially metabolic syndrome and insulin resistance, rather than a direct effect on LC risk (32). Vaccination was also significantly and positively associated with LC. This conflicts with Antonelli et al. (33), where LC symptoms were reported less frequently in infected vaccinated individuals than in infected unvaccinated individuals. However, our vaccination variable simply indicates whether a patient ever received at least one dose, without distinguishing the number of doses or timing relative to infection, and most of our cohort (first and second waves) acquired COVID-19 before Italy's vaccine rollout began in December 2020. As a result, vaccination followed rather than preceded infection.

Then, also if our findings are in line with those of the Scottish study (9), we cannot confirm their results because only a small fraction of our sample was vaccinated before the infection, and broader evidence links incomplete or absent vaccination to increased LC risk (34, 35).

Regarding the prevalence of individual symptoms of LC, the rates are consistent across the definitions at 3- and 6-months post-infection (except for hair loss), but they differ from those based on the WHO definition. For most symptoms, the adjusted prevalence differs significantly from the naïve rates, emphasizing the importance of jointly modelling selection and outcomes to estimate unbiased prevalence rates. Unlike dyspnea, for other symptoms, the effect of selection bias diminishes once observed covariates are accounted for. This suggests the presence of unmeasured factors contributing to LC prevalence in dyspnea, such as related to neurological or brain-related causes or other not measured risk factors.

Based on the adjusted prevalence, the most common LC symptoms are fatigue, followed by joint and muscle pain, and dyspnea. These findings align with Ballering et al. (10), which identified the most severe symptoms during the 3–5 months post-acute COVID-19 phase as musculoskeletal symptoms (e.g., muscle pain), sensory symptoms (e.g., anosmia or ageusia), and general symptoms (e.g., fatigue and heaviness in the limbs). The same study also highlighted the potential impact of COVID-19 on mental health, emphasizing symptoms such as anxiety, amnesia, and insomnia, supporting the hypotheses proposed in the Netherlands report. Additionally, our naïve prevalence rates for LC symptoms at 3 and 6 months closely resemble those reported in a systematic review assessing symptom persistence in COVID-19 outpatients (8).

Building on these insights, our findings suggest several concrete steps for improving both patient care and health-system responses. In clinical settings, integrating cardiovascular and metabolic comorbidities into a unified risk assessment will help identify those most vulnerable to persistent symptoms, enabling tailored follow-up such as scheduled metabolic panels, early referral to endocrinology or rehabilitation services, and enrolment in multidisciplinary LC programs that combine respiratory evaluation with nutritional counselling, graded exercise, and mental-health support. At the health-system level, adopting harmonized LC definitions and establishing dedicated surveillance infrastructure are essential to accurately measure burden and direct resources. Policymakers should consider funding specialized LC clinics, issuing clear guidance on optimal vaccination timing relative to infection, and equipping primary-care providers with streamlined follow-up protocols. Finally, planning for recovery and future pandemic preparedness must explicitly account for LC's economic impact, namely, reduced workforce productivity and increased healthcare utilization.

## 5 Conclusion

To our knowledge, this is the first study to examine the nature and prevalence of LC while accounting for both observed and unobserved confounders. Our analysis demonstrates that selection bias persists despite adjusting for known covariates, leading to significant differences between adjusted and naïve prevalence estimates. These findings highlight the critical need for rigorous methodological approaches that jointly model selection and outcome to obtain accurate prevalence estimates—an aspect frequently overlooked in LC research.

Looking ahead, future studies should embed selection-outcome frameworks, such as inverse-probability weighting or joint likelihood models, from the outset, while also capturing pre-infection baselines via electronic health records or wearable sensors to distinguish new sequelae from chronic symptoms and track real-time trajectories. Symptom inventories must broaden beyond our initial 11 items to include autonomic, neuropsychiatric, and dysautonomic manifestations, underpinned by harmonized case definitions and core outcome sets to enable global comparability. Mechanistic work pairing biomarker panels (inflammatory cytokines, autoantibodies, endothelial markers) with imaging and functional assessments will be essential for identifying therapeutic targets. Complementing these efforts, adaptive trials that stratify participants by cardiovascular or metabolic risk profiles can efficiently evaluate precision interventions, whether cardioprotective regimens or insulin-sensitizing therapies. Finally, ensuring demographic and geographic diversity, and rigorously measuring LC's impact on quality of life, mental health, and socioeconomic outcomes, will be vital for crafting equitable clinical pathways and informing public health policy.

### 5.1 Strengths and limitations

This study has several strengths, including its large sample size of hospitalized and non-hospitalized populations followed for 30 months, precise infection dates with laboratory confirmation, and inclusion of a comparison group with pre-COVID-19 comorbidities and acute-phase treatments. Unlike many studies, symptoms were monitored longitudinally during follow-up, rather than assessed only at the start of the pandemic. It is also the first study to report LC prevalence while adjusting for selection bias, pre-existing conditions, COVID-19 severity, and therapies during the acute phase.

However, the study has limitations. Despite adjustment for known covariates, residual selection bias may persist and contribute to the observed differences in adjusted prevalence estimates. We identified an association between age and LC, participants aged 40–50 years had higher odds of persistent symptoms at 3 and 6 months compared with those aged 18–30 years, which contradicts published data and likely reflects underrepresentation of younger individuals in our sample. Moreover, while we observed associations between certain medications (e.g., antiplatelets, statins, and metformin) and LC outcomes, these findings must be interpreted cautiously given the study's observational design and potential for unmeasured confounding.

The lack of pre-infection symptom data prevents us from distinguishing new LC manifestations from continuations of pre-existing conditions. Our questionnaire covered only 11 predefined symptoms, potentially omitting other clinically relevant sequelae, and asymptomatic SARS-CoV-2 infections were not captured. Finally, because our population was drawn exclusively from northern Italy, the generalizability of these findings to other geographic or healthcare settings remains uncertain.

## Data Availability

The raw data supporting the conclusions of this article will be made available by the authors, without undue reservation.
