# Supplementary material for: Estimating long COVID-19 prevalence across definitions and forms of sample selection
Source: Front Epidemiol. 2025 May 30;5:1597799. doi: 10.3389/fepid.2025.1597799 (PMC12164371; doi:10.3389/fepid.2025.1597799)
Supplement: Supplementary file 1 [file Table1.docx]

**Table 1**: Counts, percentages and Odds Ratio (OR, treated vs control) and OR significance (P)

| Covariates | Levels | Controls | Participants | Overall | OR | P |
| --- | --- | --- | --- | --- | --- | --- |
|  |  | **(N=1807)** | **(N=1537)** | **(N=3344)** |  |  |
| Age Group | [18, 30) | 64 (3.5%) | 125 (8.1%) | 189 (5.7%) | ￼ | ￼ |
| ￼ | [30,40] | 104 (5.8%) | 147 (9.6%) | 251 (7.5%) | 0.724 | ￼ |
| ￼ | (40,50] | 225 (12.5%) | 260 (16.9%) | 485 (14.5%) | 0.592 | *** |
| ￼ | (50,102] | 1414 (78.3%) | 1005 (65.4%) | 2419 (72.3%) | 0.364 | *** |
| Sex | Male | 1110 (61.4%) | 742 (48.3%) | 1852 (55.4%) | ￼ | ￼ |
| ￼ | Female | 697 (38.6%) | 795 (51.7%) | 1492 (44.6%) | 1.706 | *** |
| Obesity | 0 | 1381 (76.4%) | 1283 (83.5%) | 2664 (79.7%) | ￼ | ￼ |
| ￼ | 1 | 426 (23.6%) | 254 (16.5%) | 680 (20.3%) | 0.642 | *** |
| Lung | 0 | 1544 (85.4%) | 1364 (88.7%) | 2908 (87.0%) | ￼ | ￼ |
| ￼ | 1 | 263 (14.6%) | 173 (11.3%) | 436 (13.0%) | 0.745 | *** |
| Heart | 0 | 889 (49.2%) | 989 (64.3%) | 1878 (56.2%) | ￼ | ￼ |
| ￼ | 1 | 918 (50.8%) | 548 (35.7%) | 1466 (43.8%) | 0.537 | *** |
| Metabolic | 0 | 1233 (68.2%) | 1150 (74.8%) | 2383 (71.3%) | ￼ | ￼ |
| ￼ | 1 | 574 (31.8%) | 387 (25.2%) | 961 (28.7%) | 0.723 | *** |
| Renal | 0 | 1657 (91.7%) | 1477 (96.1%) | 3134 (93.7%) | ￼ | ￼ |
| ￼ | 1 | 150 (8.3%) | 60 (3.9%) | 210 (6.3%) | 0.449 | *** |
| Oncological | 0 | 1590 (88.0%) | 1493 (97.1%) | 3083 (92.2%) | ￼ | ￼ |
| ￼ | 1 | 217 (12.0%) | 44 (2.9%) | 261 (7.8%) | 0.216 | *** |
| Immune System | 0 | 1675 (92.7%) | 1448 (94.2%) | 3123 (93.4%) | ￼ | ￼ |
| ￼ | 1 | 132 (7.3%) | 89 (5.8%) | 221 (6.6%) | 0.780 | * |
| Hepatopathy | 0 | 1738 (96.2%) | 1510 (98.2%) | 3248 (97.1%) | ￼ | ￼ |
| ￼ | 1 | 69 (3.8%) | 27 (1.8%) | 96 (2.9%) | 0.450 | *** |
| Diabetes | 0 | 1559 (86.3%) | 1397 (90.9%) | 2956 (88.4%) | ￼ | ￼ |
| ￼ | 1 | 248 (13.7%) | 140 (9.1%) | 388 (11.6%) | 0.630 | *** |
| #Comorbidities | Mean (Std. Dev.) | 1.66 (1.40) | 1.17 (1.25) | 1.43 (1.36) | 0.755 | ******* |
| ￼ | Median [Min, Max] | 1.00 [0, 7.00] | 1.00 [0, 7.00] | 1.00 [0, 7.00] | ￼ | ￼ |

Sign levels: ^*^p<0.1; ^**^p<0.05; ^***^p<0.01

**Table 2**: Counts, percentages and Odds Ratio (OR, treated vs control) and OR significance (P)

| Covariates | Controls | Participants | Overall | OR | P |
| --- | --- | --- | --- | --- | --- |
|  | **(N=1807)** | **(N=1537)** | **(N=3344)** |  |  |
| COVID-19 Severity (WHO scale) | ￼ | ￼ | ￼ | ￼ | ￼ |
| 1- Asymptomatic COVID-19 | 256 (14.2%) | 650 (42.3%) | 906 (27.1%) | - | ￼ |
| 2- Extrapulmonary COVID-19 | 811 (44.9%) | 339 (22.1%) | 1150 (34.4%) | 0.165 | *** |
| 3- COVID-19+lung insufficiency | 490 (27.1%) | 190 (12.4%) | 680 (20.3%) | 0.153 | *** |
| 4- COVID-19+lung insufficiency+O2 support | 250 (13.8%) | 358 (23.3%) | 608 (18.2%) | 0.564 | *** |
| O2 Therapy during COVID-19 | ￼ | ￼ | ￼ | ￼ | ￼ |
| 1-NO O2 therapy | 355 (19.6%) | 818 (53.2%) | 1173 (35.1%) | - | ￼ |
| 2-Nasal cannula | 445 (24.6%) | 193 (12.6%) | 638 (19.1%) | 0.188 | *** |
| 3-Venturi mask | 410 (22.7%) | 196 (12.8%) | 606 (18.1%) | 0.207 | *** |
| 4-Reservoir mask | 75 (4.2%) | 29 (1.9%) | 104 (3.1%) | 0.168 | *** |
| 5-Non-invasive ventilation | 357 (19.8%) | 240 (15.6%) | 597 (17.9%) | 0.292 | *** |
| 6-IOT (Tracheal intubation) | 165 (9.1%) | 61 (4.0%) | 226 (6.8%) | 0.160 | *** |

O2 Therapy: Nasal cannula: low O2 flow; Venturi mask: 28-60% O2 flow; Reservoir mask: 100%O2 flow; Non-invasive ventilation: 100%O2 flow.

Sign levels: *p<0.1; **p<0.05; ***p<0.01

￼

￼

**Table 3:** Upper: ARCOVID Participants (N) and Prevalence of Long COVID-19 by definition.

Lower: ARCOVID Participants (N) and Prevalence (Prev.) of single symptoms, overall period by wave.

| Covariates | Overall period | Wave1 | Wave2 | P^^^ |
| --- | --- | --- | --- | --- |
|  | N = 1,537 | N = 476 | N =1,061 |  |
| Long COVID-19 | N (Prev.) | ￼ | ￼ | ￼ |
| LC_WHO | 1036 (67.4%) | 342 (72.0%) | 694 (65.3%) | <0.001 |
| LC_Nice | 1173 (76.3%) | 371 (78.1%) | 802 (75.5%) | <0.001 |
| LC_3m | 1232 (80.2%) | 383 (80.6%) | 849 (79.9%) | <0.001 |
| LC_6m | 1223 (79.6%) | 379 (79.8%) | 844 (79.5%) | <0.001 |
| Single symptom | N (Prev.) | ￼ | ￼ | ￼ |
| Dyspnea | 631 (41.1%) | 185 (38.9%) | 446 (42.0%) | <0.001 |
| Hair loss | 405 (26.3%) | 140 (29.5%) | 265 (24.9%) | <0.001 |
| Fatigue | 900 (58.6%) | 286 (60.2%) | 614 (57.8%) | <0.001 |
| Join Muscle Pain | 602 (39.2%) | 202 (42.5%) | 400 (37.7%) | <0.001 |
| Palpitations | 383 (24.9%) | 130 (27.4%) | 253 (23.8%) | <0.001 |
| Smell Loss | 334 (21.7%) | 140 (29.5%) | 194 (18.3%) | <0.001 |
| Taste Loss | 322 (20.9%) | 133 (27.9%) | 189 (17.8%) | 0.001 |
| Headaches | 299 (19.5%) | 104 (21.9%) | 195 (18.4%) | <0.001 |
| Anxiety | 352 (22.9%) | 116 (24.4%) | 236 (22.2%) | <0.001 |
| Insomnia | 396 (25.8%) | 96 (20.2%) | 300 (28.2%) | <0.001 |

LC: Long Covid

^Significance of differences of prevalence across waves.

￼

￼

**Table 4:** Upper: Naïve and adjusted Prevalence by LC definition and significance of the sample selection (Rho) of the bivariate Probit model. Lower: covariates and significance (P) of the selection equation

| Outcome | LC_WHO | | | LC_3m | | | LC_6m | | |
| --- | --- | --- | --- | --- | --- | --- | --- | --- | --- |
| Naïve Prev. (95% CI) | 67.4% (65.1%-69.7%) | | | 80.2% (78.2%-82.1%) | | | 79.6% (77.6%-81.6%) | | |
| Adjusted Prev. (95% CI) | 66.1% (50.1%-75.6%) | | | 81.1% (71.3%-86.7%) | | | 79.7% (71.2%-85.3%) | | |
| Rho^T^ | 0.19 (-0.03; 0.37) | | | **0.37** (0.11; 0.57) | | | **0.31** (0.06; 0.49) | | |
| Selection equation | | | | | | | | | |
|  | Coeff. | OR^⊥^ | P | Coeff. | OR^⊥^ | P | Coeff. | OR^⊥^ | P |
| Constant | 0.603 | 2.671 | *** | 0.796 | 3.696 | *** | 0.797 | 3.704 | *** |
| Wave (2 vs 1) | ￼ | ￼ | ￼ | -0.119 | 0.821 | ** | -0.119 | 0.820 | ** |
| Oncological | -0.825 | 0.243 | *** | -0.824 | 0.243 | *** | -0.826 | 0.243 | *** |
| Renal | -0.322 | 0.579 | *** | -0.322 | 0.578 | *** | -0.323 | 0.577 | *** |
| Lung | -0.105 | 0.833 | . | -0.118 | 0.816 | * | -0.117 | 0.817 | * |
| Immune system | -0.153 | 0.763 | . | -0.141 | 0.781 | . | -0.146 | 0.775 | ￼ |
| O2_therapy - Nasal cannula | -1.010 | 0.196 | *** | -0.991 | 0.201 | *** | -0.991 | 0.201 | *** |
| O2_therapy - Venturi mask | -0.932 | 0.219 | *** | -0.908 | 0.229 | *** | -0.91 | 0.228 | *** |
| O2_therapy - Reservoir mask | -1.083 | 0.176 | *** | -1.089 | 0.174 | *** | -1.076 | 0.177 | *** |
| O2_therapy -  Non invasive ventilation | -0.758 | 0.292 | *** | -0.739 | 0.301 | *** | -0.736 | 0.302 | *** |
| O2_therapy - IOT  (Tracheal intubation) | -1.153 | 0.152 | *** | -1.137 | 0.156 | *** | -1.138 | 0.156 | *** |

O2 Therapy: Nasal cannula: low O2 flow; Venturi mask: 28-60% O2 flow; Reservoir mask: 100%O2 flow; Non-invasive ventilation: 100%O2 flow.

T Bold indicates significant sample selection effect (significant Rho estimate).

⊥OR estimated using a bivariate Logit model.

Sign levels: “.” p<0.1; “*“p<0.05; “**“p<0.01; “***“p<0.001.

￼

￼

**Table 5:** Outcome equation for event “Long COVID-19” by LC definition: coefficient and significance (P) of the bivariate Probit model and Odds ratio (OR) of the bivariate Logit model

| Covariates | LC_WHO | | | LC_3m | | | LC_6m | | |
| --- | --- | --- | --- | --- | --- | --- | --- | --- | --- |
| ￼ | Coeff. | P | OR^^^ | Coeff. | P | OR^^^ | Coeff. | P | OR^^^ |
| Constant | 0.048 | 0.874 | 1.074 | 0.432 | . | 1.942 | 0.300 | . | 1.541 |
| Sex Female | 0.474 | *** | 2.165 | 0.510 | *** | 2.351 | 0.515 | *** | 2.379 |
| Heart disease | 0.196 | ** | 1.371 | ￼ | ￼ | ￼ | ￼ | ￼ | ￼ |
| Metabolic disease | ￼ | ￼ | ￼ | 0.310 | *** | 1.629 | 0.288 | *** | 1.587 |
| Age group [30-40] | ￼ | ￼ | ￼ | 0.280 | * | 1.580 | 0.237 | . | 1.482 |
| Age group (40-50] | ￼ | ￼ | ￼ | 0.370 | ** | 1.847 | 0.334 | ** | 1.748 |
| Age group (50-102] | ￼ | ￼ | ￼ | 0.263 | * | 1.520 | 0.256 | * | 1.510 |
| Age group [18-30) | Reference | ￼ | ￼ | - | ￼ | ￼ | - | ￼ | ￼ |
| Therapies during COVID-19 | | ￼ | ￼ | ￼ | ￼ | ￼ | ￼ | ￼ | ￼ |
| Antiaggregant | -0.273 | ** | 0.640 | -0.231 | * | 0.689 | ￼ | ￼ | ￼ |
| Statin | ￼ | ￼ | ￼ | -0.215 | . | 0.716 | -0.299 | ** | 0.616 |
| Hypoglycaemics | -0.210 | . | 0.706 | -0.310 | ** | 0.602 | -0.323 | ** | 0.593 |
| Antiretroviral | -0.541 | *** | 0.410 | -0.616 | *** | 0.364 | -0.599 | *** | 0.373 |
| HCQ | 0.340 | *** | 1.752 | 0.236 | * | 1.491 | 0.251 | ** | 1.540 |
| Antibiotic | 0.235 | *** | 1.474 | 0.175 | ** | 1.336 | 0.178 | ** | 1.356 |
| Vaccine | 0.094 | . | 1.167 | 0.137 | ** | 1.254 | 0.143 | ** | 1.270 |

^^^estimated using a bivariate Logit model. Sign levels: “.” p<0.1; “*“p<0.05; “**“p<0.01; “***“p<0.001.
